# Empirical bayes analysis of sequencing-based transcriptional profiling without replicates

**DOI:** 10.1186/1471-2105-11-564

**Published:** 2010-11-16

**Authors:** Zhijin Wu, Bethany D Jenkins, Tatiana A Rynearson, Sonya T Dyhrman, Mak A Saito, Melissa Mercier, LeAnn P Whitney

**Affiliations:** 1 Center for Statistical Sciences and Department of Community Health, Box G-121S-7, Brown University, Providence RI 02912, USA; 2 Department of Cell and Molecular Biology The University of Rhode Island, 120 Flagg Road, Kingston, RI 02881, USA; 3 The Graduate School of Oceanography, University of Rhode Island, South Ferry Road, Narragansett, RI 02882, USA; 4 Biology Department, Woods Hole Oceanographic Institution, Woods Hole MA 02543, USA; 5 Marine Chemistry and Geochemistry Department, Woods Hole Oceanographic Institution, 360 Woods Hole Rd, Woods Hole MA 02543, USA

## Abstract

**Background:**

Recent technological advancements have made high throughput sequencing an increasingly popular approach for transcriptome analysis. Advantages of sequencing-based transcriptional profiling over microarrays have been reported, including lower technical variability. However, advances in technology do not remove biological variation between replicates and this variation is often neglected in many analyses.

**Results:**

We propose an empirical Bayes method, titled Analysis of Sequence Counts (ASC), to detect differential expression based on sequencing technology. ASC borrows information across sequences to establish prior distribution of sample variation, so that biological variation can be accounted for even when replicates are not available. Compared to current approaches that simply tests for equality of proportions in two samples, ASC is less biased towards highly expressed sequences and can identify more genes with a greater log fold change at lower overall abundance.

**Conclusions:**

ASC unifies the biological and statistical significance of differential expression by estimating the posterior mean of log fold change and estimating false discovery rates based on the posterior mean. The implementation in R is available at http://www.stat.brown.edu/Zwu/research.aspx.

## Background

Recent technological advancements have made high throughput sequencing an increasingly popular approach for transcriptome analysis. Unlike microarrays, enumeration of transcript abundance with sequencing technology is based on direct counts of transcripts rather than relying on hybridization to probes. This has reduced the noise caused by cross-hybridization and the bias caused by the variation in probe binding efficiency. Sequencing-based transcription profiling does have other challenges. For example, whole transcript analysis produces data with transcript length bias [1]. Other biases, including GC content, have also been reported [2]. Nonetheless, sequencing based expression analysis has been shown to be more robust and have higher resolution compared to microarray platforms [3]. Some researchers have predicted that it will eventually replace microarrays as the major platform for monitoring gene expression [4]. The importance of replicates is well recognized in microarray analysis [5] and it is now standard practice to include biological replicates under each experimental condition. However, as of now, sequencing-based gene expression studies often do not include replicates [6-8], posing the question of whether the biological variation is, or can be, adequately addressed.

For illustration, we use data from Illumina Digital Gene Expression (DGE) tag profiling in this paper. However, our statistical methodology, and its implementation in R, are general for all sequencing-based technologies that quantify gene expression as counts instead of continuous measurements such as probe intensity in microarrays. In DGE, the 3' end of transcripts with a poly-A tail are captured by beads coated with oligo dT. Two restriction enzymes, NlaIII and Mmel are used to digest the captured transcripts, generating a 21-base fragment starting at the most 3' NlaIII site. The 21-base fragments are sequenced to quantify the transcriptome. Consider two samples in a comparison and let *X*_1 _and *X*_2 _be the counts of a particular sequence tag in the two samples. The most common approach is to consider the counts as a realization of binomial distribution *B*(*N_i_*, *π_i_*), *i *= 1,2. where *N_i _*is the total number of sequences in a sample, representing sequencing depth. A statistical test for *π*_1 _= *π*_2 _can be conducted. The classical Z-test using the Gaussian approximation to the binomial distribution is proposed for the Serial Analysis of Gene Expression (SAGE) data [9,10] and recently applied to DGE and other sequencing data [11-13], and Fisher's exact test has also been proposed [14]. In other technologies, sequence counts may have to be combined at either the exon or full transcript level to form the counts *X*_1 _and *X*_2_.

The test for *H*_0 _: *π*_1 _= *π*_2 _can be performed without replicates. However, rejection of the *H*_0 _hypothesis simply implies difference between the two samples. Unless the proportion of a gene in the transcriptome is the same for all samples under the same condition (lack of within-class variation), we can not generalize the difference between two samples to the difference between two classes. The within-class biological variation among replicates leads to over dispersion in Binomial or Poisson models. Models accounting for over dispersion, such as a beta-binomial, have been introduced for the analysis of SAGE data when several replicates within each class are available [15,16]. Robinson and Smyth [17] use a negative binomial model and squeeze tag-wise dispersion towards a common dispersion, estimated using all tags, with a weighted likelihood approach that yields an EB-like solution. edgeR [18], a Bioconductor package implementing this method, has been applied to both DGE and RNA-seq data with replicates. However, since replicates are still rare in high throughput sequencing, many researchers have been relying on simple tests of equal proportion with multiple testing correction. Another drawback in some current analysis methods, especially those applied to the no-replicate situation, is the use of Gaussian approximation of binomial distribution [11,19], which does not work well with data that include low count numbers. In transcriptome analysis, due to the depth of sequencing, the majority of genes have low counts. Relying on Gaussian distribution often gives highly expressed genes favorable statistical power, such that genes that have a lower expression but exhibit greater extent of differential expression between samples are less likely to be discovered.

In this paper we present an empirical Bayes method, titled Analysis of Sequence Counts (ASC), to estimate the log fold change of transcription between two samples. We borrow information across sequences to estimate the hyper parameters representing the normal biological variation among replicates and the distribution of a transcriptome. The statistical model does not rely on Gaussian approximation of the binomial distribution for all tags and requires no special treatment of 0 counts. Differential expression is computed in the form of a shrinkage estimate of log fold change. This estimate is the basis for ranking genes. We also compute the posterior probability that the log fold change is greater than a biologically relevant threshold chosen by the user. In contrast to sorting genes simply by p-values, we focus on the biological significance (represented by the posterior expectation of log fold change) and provide uncertainty measure in the form of posterior probability.

### Modeling biological variation

**Figure 1:**
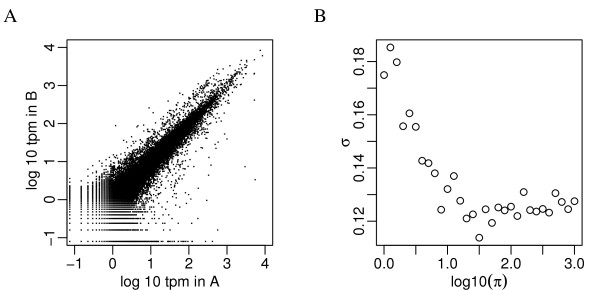
**Scatter plot of the log_10 _rpm in two samples**. A. Scatter plot of the log_10 _rpm in two samples. The greater spread at lower counts suggests higher variance. B. Sample variance of log_10 _rpm from a simulation with constant biological variation shows that the increased sample variance may be caused by poor approximation of a Gaussian distribution to a Binomial distribution for sparse counts. To create the simulation dataset, we sampled log(*π*_0_) from observed average log counts and created log(*π*_1_) = log(*π*_0_) + *δ/*2 and log(*π*_2_) = log(*π*_0_) - *δ/*2 where *δ *~ *N *(0, *σ*^2^). In this plot *σ *= 0.122, as estimated in the example data.

**Figure 2:**
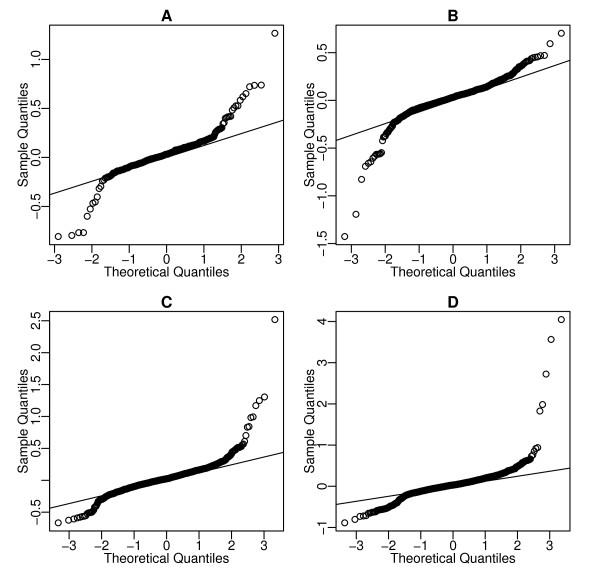
**Quantile quantile (QQ) plots of the differences of log rpm (log(*p*_1_) - log(*p*_2_)) confirming the Gaussian distribution as a reasonable approximation of the biological variation**. A.QQ plots of log(*p*_1_) - log(*p*_2_) for genes with average log rpm within 2 ± 0.05. B) For genes with average log rpm within 1.6 ± 0.05 C) For genes with average log rpm within 1.2 ± 0.05 D) For genes with average log rpm within 0.8 ± 0.05. The straight lines in all figures have a slope 0.28, suggesting that biological variation does not depend on average rpm.

It has been reported that the noise in gene expression by sequencing depends on expression level as observed in microarray data [19]. It has been widely observed that the scatter plot of the log reads-per-million (rpm) between samples have wider spread for lower average counts, as shown in Figure 1A. This is shown by the relationship between the empirical variance of log rpm across replicates and the average of log rpm. For example, Stolovitzky et al [19] binned genes whose average rpm are closest and computed sample standard deviation (SD) of genes within each bin, and reported higher SD in bins with smaller rpm. However, the sample SD is only expected to be a good estimate of the biological variation of log expression when the Gaussian approximation works well. We conducted a simulation in which the biological variation of log expected rpm is constant, and the observed rpm is generated from a Poisson distribution. The sample SD of log rpm, as shown in Figure 1B, also appears to be inflated for low expression genes. This demonstrates that the inflated variance in observed log rpm does not necessarily imply higher biological variation, but often is a result of poor approximation of a Binomial random variable by a Gaussian distribution. Figure 2 shows that the quantile-quantile plots of the differences of observed log rpm (i.e., the observed log fold change) in *T. pseudonana *data (see Methods) at various average expression levels. The straight lines in all plots have the same slope, suggesting stable biological variation. The fact that most of the points stay on the straight line also confirms that a Gaussian approximation is a reasonable choice for the biological variation.

### Distribution of expression levels in a transcriptome

**Figure 3:**
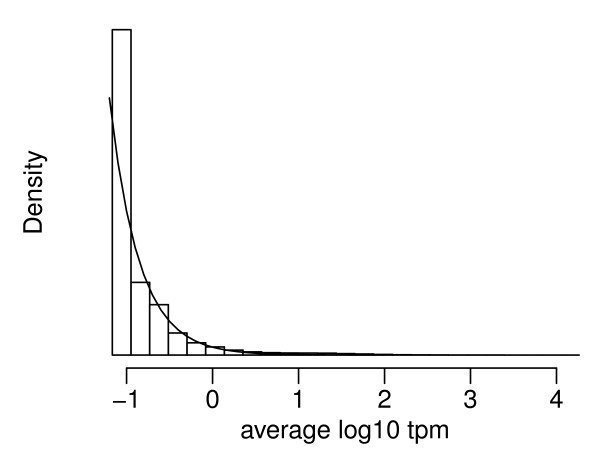
**Histogram of the average log_10 _rpm between the A and B samples**. Histogram of the average log_10 _rpm between the A and B samples. The smooth curve shows the probability density function of the fitted shifted exponential distribution.

As observed in both microarray data and sequencing-based transcriptome profiling, genes can differ by orders of magnitude in their expression levels, ranging from less than 1 per million to thousands per million and the majority of genes have relatively low counts. Tags with 0 counts cause problems in statistical analyses that take a direct log transformation and some investigators have had to develop special treatments for those genes [19]. In Figure 3 we show the empirical distribution of the average log rpm, defined as [log_10_(*x*_1 _∧ 0.5)*/N*_1 _+ log_10_(*x*_2 _∧ 0.5)*/N*_2_] */*2. This is a highly skewed distribution even in the log scale. The skewness motivates us to use a shifted exponential distribution as the prior distribution for the average expression level.

## Results and Discussion

**Figure 4:**
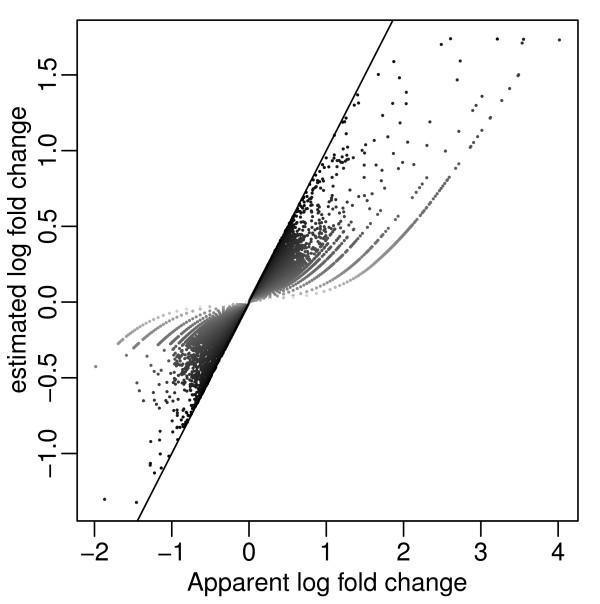
**Shrinkage estimate of log fold change**. Shrinkage estimate of log fold change from ASC plotted against apparent log fold change for all genes. The apparent log fold change is defined as $\log_{10}\{(x_{1}^{*}/N_{1})/(x_{2}^{*}/N_{2})\}$ where x* = *x *∧ 0.5 to avoid logarithm of 0 counts. The darkness of each point reflects the average rpm, $[\log_{10}(x_{1}^{*}/N_{1})+log_{10}(x_{2}^{*}/N_{2})]/2$, of a gene, with the darkest being the highest average rpm. The straight line is the identity line representing no shrinkage. The genes with lighter gray have lower average rpm and are shrunk more.

We applied ASC to transcription profiles of the diatom *Thalassiosira pseudonana *under two culturing conditions, measured by DGE, and computed the posterior expectation of log fold change for all genes. Figure 4 compares the shrinkage estimate with the "apparent log fold change" based on sample proportions. In order to display all tags including those with count 0 in log scale, we define the *apparent log ratio *as $\log_{10}\left[\frac{(x_{1}\wedge 0.5)/N_{1}}{(x_{2}\wedge 0.5)/N_{2}}\right]$. We also define the average proportion for each gene as the geometric mean of the two proportions, with similar adjustment at 0. This adjustment at 0 is for the completeness of visual presentation, and is not done in ASC analysis. The estimated fold change from genes with high counts are almost the same as the apparent fold change, but genes with sparse counts are shrunk more aggressively (Figure 4). This is a desirable property since the coefficient of variation of binomial distribution decreases with the expectation. This implies that when the expected count is small, it is much easier to produce counts with apparent large fold changes.

**Figure 5:**
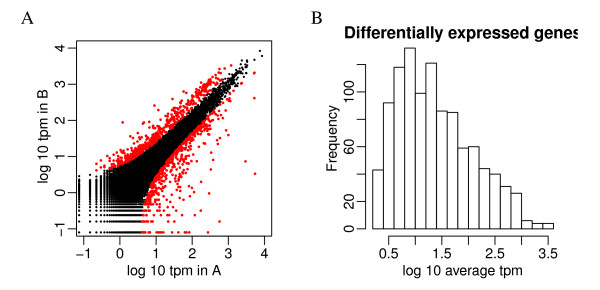
**Differential expressed genes identified by ASC**. A. Scatter plot of the log_10 _rpm in two samples. Differentially expressed genes with posterior probability of fold change greater than 2 are highlighted in red. B. Distribution of average rpm for the highlighted differentially expressed genes.

We estimated the posterior mean of log fold change and the posterior probability that there is greater than two fold change for a given tag. There are 1050 genes with posterior probability greater than 0.9 that the fold change is greater than 2. The average log rpm of those tags spread from less than 0.23 (1.7 rpm) to 3.6 (10,000 rpm) and most have approximately 1 (10 rpm). Figure 5A highlights these genes in a scatter plot of observed log rpm. F5B shows the distribution of average rpm of these genes, suggesting that majority of genes displaying differential expression by ASC analysis have moderate counts, even though we have aggressively shrunk the estimate of log fold change for tags with low counts.

### Comparison with other methods

**Figure 6:**
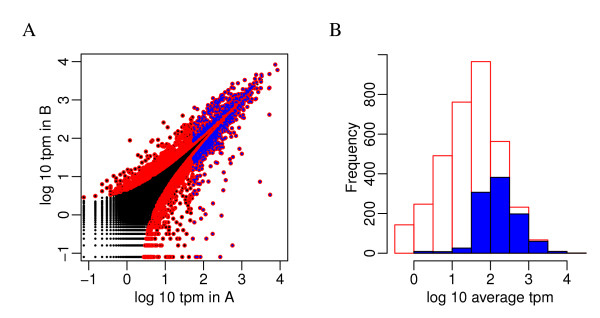
**Differential expressed genes identified by Z-test**. A. Scatter plot of the log_10 _rpm in the A and B samples. Differentially expressed genes with Bonferonni adjusted p-value less than 0.05 highlighted in red and the smallest 1000 of which highlighted in blue. B. Distribution of average rpm for the highlighted red or blue genes.

All of the genes identified as differentially expressed by ASC have very small p-values if a simple test of equal proportions is performed. In fact, a simple Z-test identifies 3479 differentially genes at significance level 0.05 with Bonforonni correction, as highlighted in red in Figure 6A. Fisher's exact test gives almost the same results except adding a few more genes with less total tag counts. We highlight the top 1000 genes with smallest p-values in blue, and it is clear that genes with lower average expression are not identified. Figure 6B shows that the majority of genes identified to have differential expression have much higher average counts compared to those identified by ASC. We have also applied a software, DGEseq [11], recently developed specifically for the analysis of digital gene expression data as in this example. DGEseq identified more than 7000 genes with estimated FDR less than 0.01, and also favors transcripts with higher average rpm. The results are included in additional file 1, Figure S1.

ASC clearly prioritizes genes differently from Z test or DGEseq and finds more genes with modest expression but greater fold change as differentially expressed. In order to show that the top ranked genes in ASC are associated with higher biological significance, we obtained DGE data from an experiment comparing expression from two genotypes with 4 replicates each [3] (GSE10782). Hoen et al [3] compute Bayes Error using SAGE BetaBin [16], a method that takes into account of biological variation between replicates. The Bayes Error in SAGE BetaBin represents the "superposition" between the estimated posterior distributions of the classes in comparison and is used to rank the genes for differential expression. To evaluate how various methods work when replicates are not available, we choose the first sample of each genotype and obtained lists of the top 1000 tags and compared the Bayes Error for those tags based on the full data. Among the top 1000 tags found by ASC, 320 have an estimated Bayes Error of approximately 0, significantly more than those found by the other statistics (Table 1).

**Table 1:** Overlap between the top 1000 genes identified by different methods and the SAGE BetaBin ranking.

Bayes Error by SAGE BetaBin	ASC	DGEseq	Z-test	Fisher's exact test	EdgeR
≈ 0	320	189	178	180	259
≤ 0.01	391	254	242	244	332
≤ 0.05	516	404	397	398	436

The Bayes error is computed using SAGE BetaBin [16] using all replicates. The top 1000 genes by each methods in the model is identified by using only the first sample in Wild-type or mutant group in [3] (GSM272105 and GSM272106).

We have also used edgeR [18], a moderated statistical test for sequencing data with replicates [17,20], to analyze the full dataset. For the no replicate case, we first used the two samples as replicates to estimate the dispersion parameter in edgeR and then estimated differential expression given that dispersion. We compared the overlap between the top ranked differentially expressed genes using edgeR on the full data (using edgeR) and top ranked genes from the data without replicates (using ASC, edgeR, DGEseq, Z and fisher's exact test). The agreement between ASC and full data edgeR is almost identical to the agreement between no-replicate and full data edgeR, while the latter is expected since it is based on the same methodology. About a third of the top 100 genes identified in the full data are recovered in the top 100 genes ranked by ASC. In contrast, less than 2 of the top 100 genes from full data analysis made to the top 100 list by the other methods. The comparison is summarized in Table 2.

**Table 2:** Overlap between the top 100 or top 1000 differentially expressed genes identified by edgeR on full data and by other statistics on data without replicate

edgeR on full data	Without replicates				
	**ASC**	**edgeR**	**DGEseq**	**Z**	**Fisher**
top 100	33	37	2	1	1
top 1000	260	263	89	82	86

**Figure 7:**
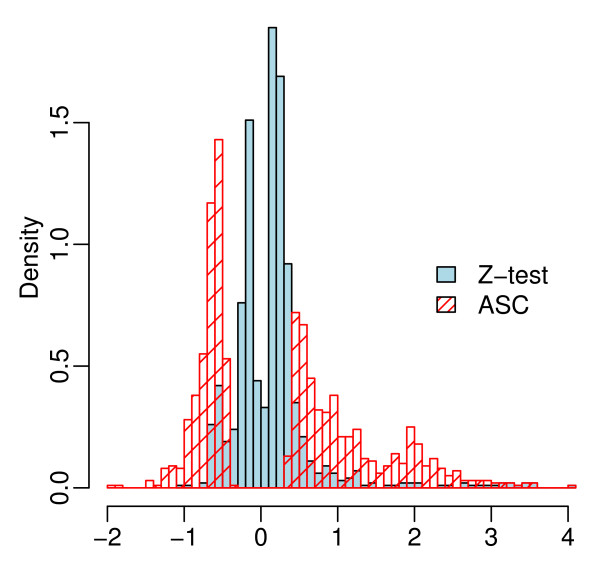
**Histogram of the apparent fold change of the top 1000 genes found by Z-test or ASC**.

Why is there so little overlap between the top genes by Z-test on two sample comparison and the top genes from edgeR analysis on the full data set? Strikingly, many genes with extreme p-values in a Z-test have small fold changes. This is because there is greater statistical power to detect even subtle changes in gene expression when the counts are higher. From the Gaussian approximation to the sample proportion $p|\pi \dot{\sim }N(\pi ,\pi (1-\pi )/N)$, we have for large *N*_*π*_, the log sample proportion is also approximately Gaussian, $\log(p)|\pi \dot{\sim }N\{\log(\pi )-(1-\pi )/(2N\pi ),(1-\pi )/(N\pi )\}$. Since the expected counts *N*_*π *_varies greatly from a few to over a hundred thousand, and the variance of log sample proportion decreases sharply with the increase of expected counts, it is clear that statistical power is biased towards genes with higher counts. This also causes the bias of higher power towards longer transcripts in full transcript analysis. An extreme p-value in such a test only suggests that the proportions of a transcript is significantly different between the two samples of comparison, not whether the difference is beyond what is reasonable between biological replicates. Figure 7 overlays the distribution of apparent log fold changes for the top 1000 genes identified by either simple Z-test or ASC. Since ASC identifies a gene as differentially expressed only when a shrinkage estimate of log fold change is above certain level, the apparent log fold change from sample proportions are always away from 0. On the other hand, a simple Z-test can identify many genes with very small changes. It appears that DGEseq fails to adequately account for biological variation and the results from DGEseq are very similar to that from a Z-test (Additional file 2, Figure S2).

## Discussion

We present a simple hierarchical model for sequencing-based gene expression data (e.g. DGE, RNAseq ect.) that provides a shrinkage estimate of differential expression in the form of posterior mean of log fold change. Even in experiments lacking replicates, we take advantage of the large number of sequences quantified in the same experiment and establish a prior distribution of difference between conditions. The differential expression of a gene is evaluated based on the posterior expectation of log fold change. This estimate takes into account the increased uncertainty for genes with smaller counts (demonstrated by more aggressive shrinking in Figure 4) yet still allows the identification of differential expression among genes with lower expression. Our measure of statistical uncertainty is the posterior probability that the differential expression is beyond a given threshold, thus the inference on differential expression avoids the problem of conflicting "statistical significance" versus "biological significance" seen in Z-tests.

It is not uncommon to use hierarchical models for gene expression data. Several models used in microarray data analysis [21,22] add another level of hierarchy by assuming that only a fraction of genes may have been affected by any treatment, and the rest have absolutely no change. Therefore, *δ*|*Z *= 1 ~ *N*(0, *τ*^2^) and *δ *= 0|*Z *= 0 with *P*(*Z *= 1) = *p*_0_. This essentially assumes that the prior distribution of *δ *is a zero-inflated Gaussian distribution. We show that using a simple Gaussian prior provides good shrinkage without the extra layer of hierarchy, which greatly simplifies computation.

In biological terms, our model means that the mean gene expression levels between two populations are never absolutely equal for any gene. However, the difference for most genes are small. We use posterior expectation as the estimate of the magnitude of difference. McCarthy and Smyth [23] showed that testing the differential expression relative to a biologically meaningful threshold identifies more biologically meaningful genes. We take a similar approach and estimate the posterior probability that the differential expression is greater than a threshold. Therefore, we avoid genes with very subtle differential expression even if that difference is statistically significant between the two samples in comparison. The genes identified by ASC include those that are modestly expressed as well as highly expressed.

## Methods

### DGE data generation

The diatom *Thalassiosira pseudonana *(Strain 1335 from the Center for the Culture of Marine Phytoplankton) was grown axenically in 24 hr light at 14°C in f/2 media [24,25] made with Sargasso Sea water, Treatments consisting of phosphorus-limited medium (0.4 *μ*M PO_4_) and phosphorus-replete medium (36 *μ*M PO_4_) were grown in triplicate and are herein referred to as treatments A and B, respectively. Equal volumes of cell biomass from each replicate were pooled for the A or B treatments 96 hours after inoculation and harvested by gentle filtration. Filters were immediately frozen in liquid nitrogen and stored at -80°C.

Total RNA was extracted using the RNeasy Midi Kit (Qiagen), following the manufacturer's instructions with the following changes: RNA samples were processed with Qiashredder columns (Qiagen) to remove large cellular material and DNA was removed with an on-column DNAase digestion using RNase-free DNAase (Qiagen). A second DNA removal step was conducted using the Turbo DNA-free kit (Ambion, Austin, TX, USA)[B1]. The RNA was quantified in triplicate using the Mx3005 Quantitative PCR System (Stratagene) and the Quant-iT RiboGreen RNA Assay Kit (Invitrogen) and was analyzed for integrity by gel electrophoresis. Total RNA was sent to Illumina (Hayward, CA) and they constructed digital gene expression (DGE) libraries with NlaIII tags following their protocol. Sequencing libraries for NlaIII digested tags were constructed by Illumina and sequenced on their Genome Analyzer. 12,525,833 tags were sequenced from the A library and 13,431,745 tags were sequenced from the B library.

### Hierarchical model for gene counts

For each transcript, we assume the observed sequence counts follow a Binomial distribution given its expected expression under a biological condition. For a sequencing run that yields total count *N *for all sequence fragments, the expected count for gene *i *is expressed as *Nπ *where *π *is the expected proportion of this gene in the transcriptome. For two samples in the comparison, we observe the counts *x*_1 _and *x*_2 _while

$$\begin{array}{l} x_{1}=N_{1}p_{1}|\pi_{1}\sim Binomial(N_{1},\pi_{1})\\ x_{2}=N_{2}p_{2}|\pi_{2}\sim Binomial(N_{2},\pi_{2}) \end{array}$$

Many researchers simply test *π*_1 _= *π*_2 _and perform a Bonferonni correction to account for multiple testing. We reparametrize *π*_1 _and *π*_2 _as follows:

$$\begin{array}{l} \log(\pi_{1})=\lambda +\delta /2\\ \log(\pi_{2})=\lambda -\delta /2 \end{array}$$

Here *δ *has the interpretation of log fold change in gene expression, and *λ *is a nuisance parameter representing the average (log) expression.

We assume prior distributions

$$\begin{array}{c} \delta |\lambda \sim N(0,\tau^{2}(\lambda ))\\ \lambda \sim Exp(\alpha ,\lambda_{0}) \end{array}$$

where *Exp *represents shifted exponential distribution with rate *α *and shift *λ*_0_.

The posterior distribution of the differential expression is therefore

p(δ|x)∝∫p(x|λ,δ)p(δ|λ)p(λ)dλ.

We obtain the posterior mean $\tilde{\delta }=E[\delta |x]$ given the gene counts as an estimate of differential expression. We refer to $\tilde{\delta }$ as the shrinkage estimate of log fold change, which is sufficient to rank the genes. To evaluate the statistical significance, we compute the posterior probability P(|*δ*| > Δ_0_|**x**), where Δ_0 _is a user-defined effect size of biological significance. There is no closed form expression for the posterior distribution and we use numerical integration for the evaluation of the posterior mean and probability.

### Estimation of hyper parameters

The observed log rpm has a very skewed distribution, motivating us to use a distribution with exponential decay. But the location of this distribution is shifted compared to exponential distribution with an unknown lower bound. One advantage of the exponential distribution is the closed form expression of its cumulative density function. For *λ *~ *Exp*(*α*, *λ*_0_), $F(\lambda )=\text{1}-e^{-a(}{}^{\lambda -\lambda_{0})}$. From the sequence counts, we first compute average log rpm between the two conditions and use these to obtain the empirical CDF $\hat{F}$. Thus for two quantiles *q*_1_, *q*_2_, we can obtain empirical quantiles and $\lambda_{1}={\hat{F}}^{-1}(q_{1})$ and $\lambda_{2}={\hat{F}}^{-1}(q_{2})$. Solving the equations

(1) $$\begin{array}{l} q_{1}=1-e^{-a(\lambda_{1}-\lambda_{0})}\\ q_{2}=1-e^{-a(\lambda_{2}-\lambda_{0})} \end{array}$$

gives estimates$\left\{ \begin{array}{l} \hat{a}=-[\log(1-q_{1})-\log(1-q_{2})]/(\lambda_{1}-\lambda_{2})\\ {\hat{\lambda }}_{0}=\lambda_{1}+\log(1-q_{1})/\hat{\alpha } \end{array}\right.$

We can also use the method of moments to estimate the rate without knowing the shift parameter since the conditional expectation also has a closed from, due to the lack of memory property. For a given 0 <*q *< 1,

$$E[\lambda |\lambda >{\hat{F}}^{-1}(q)]={\hat{F}}^{-1}(q)+1/\alpha .$$

Thus we can estimate *α *as $1/\{{\bar{X}}_{X>{\hat{F}}^{-1}(q)}-{\hat{F}}^{-1}(q)\}$. Our default setting is *q*_1 _= 0.8 and *q*_2 _= 0.9. The posterior mean *E*[*δ*|**x**] is not sensitive to the choice of *q*_1_, *q*_2 _(Additional file 3, Figure S3). Again, due to the lack of memory property, the probability density of shifted exponential distributions with the same rate are proportional, thus the value of *λ*_0 _does not affect the posterior distribution and does not need to be estimated.

To estimate *τ*, the parameter representing the biological variation among replicates, we borrow information across genes. Although for any given gene we only observe one total count under each condition, and thus the true differential expression and the biological variation cannot be identified, we assume that the majority of genes are not affected by the treatment, an assumption found to be reasonable in microarray data in many experiments. We can model *τ *as a function of *λ*, but Figure 2 suggests that the biological variation is rather constant across expression levels. Thus we estimate one global parameter *τ*. We start with the Gaussian approximation of the Binomial model $p_{1}|\pi_{1}=x_{1}/N_{1}\dot{\sim }N(\pi_{1},\pi_{1}(1-\pi_{1})/N_{1})$. Since the total counts *N *are usually a very large integer, we also have, approximately, $\log(p_{1})|\pi_{1}\dot{\sim }N\{\log(\pi_{1})-(1-\pi_{1})/(2N_{1}\pi_{1}),(1-\pi_{1})/(N_{1}\pi_{1})\}$. The variance of log(*p*_1_) decreases with rate of 1/*N *as *π *increases and becomes negligible compared to biological variation. Thus we simply estimate *τ *from the differences of log rpm with the highest average log rpm. We use inter quartile range instead of sample standard deviation to avoid influence of genes with extreme differential expression. $\hat{\tau }=IQR[\log(p_{1})-\log(p_{2})]/IQR[N(0,1)]$. In practice we use total counts above 1000 and this allows us to have several thousand genes (over 4000 in our example) for the estimation.

## Authors' contributions

ZW developed the statistical methodology, in consultation with BDJ and TAR, and drafted the manuscript. BDJ, TAR, STD and MAS designed the study that generated the DGE data, and contributed to writing the manuscript. MM and LPW performed experiments that generated the DGE data. All authors have read and approved the final manuscript.

## Supplementary Material

Additional file 1 **Figure S1**. A. Scatter plot of the log_10 _rpm in the A and B samples. Differentially expressed genes identified by DGEseq with estimated q-value (Storey FDR) less than 0.01 highlighted in red and the genes with smallest q-value 1000 of which highlighted in blue. B. Distribution of average rpm for the highlighted red or blue genes. Click here for file

Additional file 2 **Figure S2**. Histogram of the apparent fold change of the top 1000 genes found by DGEseq or ASC. Click here for file

Additional file 3 **Figure S3**. Sensitivity of $\hat{\delta }$ to hyper-parameter estimation. Scatter plot of ${\hat{\delta }}_{1}$ and ${\hat{\delta }}_{1}$, based on *q*_1 _= 0.8, *q*_2 _= 0.9 and *q*_1 _= .9, *q*_2 _= 0.95, respectively. The maximum difference in estimated fold change is less than 0.04, indicating that $\hat{\delta }$ is not sensitive to the choice of *q*. Click here for file
