# Supplementary figures and images for: Empirical bayes analysis of sequencing-based transcriptional profiling without replicates
Source: BMC Bioinformatics. 2010 Nov 16;11:564. doi: 10.1186/1471-2105-11-564 (PMC3098101; doi:10.1186/1471-2105-11-564)

**apparent log fold change of top 1000 gene**

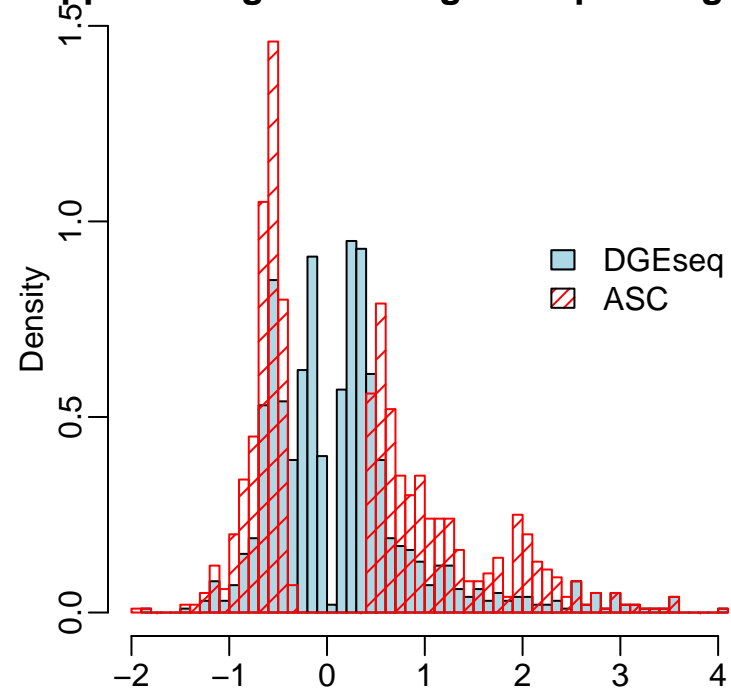

Supplement: Additional file 2 — Figure S2. Histogram of the apparent fold change of the top 1000 genes found by DGEseq or ASC. [file 1471-2105-11-564-S2.PDF]
